# Barrier Immune Effectors Are Maintained during Transition from Nurse to Forager in the Honey Bee

**DOI:** 10.1371/journal.pone.0054097

**Published:** 2013-01-08

**Authors:** Jamal M. Jefferson, Hilary A. Dolstad, Meera D. Sivalingam, Jonathan W. Snow

**Affiliations:** Biology Department, Williams College, Williamstown, Massachusetts, United States of America; Arizona State University, United States of America

## Abstract

Foragers facilitate horizontal pathogen transmission in honey bee colonies, yet their systemic immune function wanes during transition to this life stage. In general, the insect immune system can be categorized into mechanisms operating at both the barrier epithelial surfaces and at the systemic level. As proposed by the intergenerational transfer theory of aging, such immunosenescence may result from changes in group resource allocation. Yet, the relative influence of pathogen transmission and resource allocation on immune function in bees from different stages has not been examined in the context of barrier immunity. We find that expression levels of antimicrobial peptides (AMPs) in honey bee barrier epithelia of the digestive tract do not follow a life stage-dependent decrease. In addition, correlation of AMP transcript abundance with microbe levels reveals a number of microbe-associated changes in AMPs levels that are equivalent between nurses and foragers. These results favor a model in which barrier effectors are maintained in foragers as a first line of defense, while systemic immune effectors are dismantled to optimize hive-level resources. These findings have important implications for our understanding of immunosenescence in honey bees and other social insects.

## Introduction

Disease caused by infectious agents is a major selective pressure on honey bees [1]. Chronic increases in individual mortality and morbidity may lead directly to hive collapse or cause long-term reductions hive-level fitness due to loss of productivity [2], [3]. Another important consideration for hive productivity is the allocation of limited resources to the individuals and processes most likely to benefit the hive. In the context of the age-based structure of honey bee society, known as age polyethism [4], one strategy to achieve this goal is proposed by the intergenerational transfer theory of aging [5], [6]. This theory predicts that allocation of group resources to the non–reproductive individuals, such as workers, will be governed by the amount of resource transfers each individual is likely to provide to the group. Consequently, it would benefit the hive to reduce energy expended on foragers, the individuals with the least remaining lifespan in which to contribute to the colony. In correlation, significant evidence suggests that a reduction in nutrition provided to bees is partially responsible for the transition to the forager state and part of ongoing membership in the forager caste [6], [7], [8]. Importantly, foragers as a group contribute an indispensible role to colony health through their unique set of intergenerational and energetically costly transfers as the sole gatherers of food, water, and propolis for the colony. At the level of the individual forager, reduced energy input manifests itself as a loss of robustness in certain physiological functions and the molecular pathways underlying them. In fact, foragers exhibit features of senescence in multiple physiological systems [9]. As mounting an immune response is energetically costly, age-dependent loss of immune function in the form of immunosenescence might be beneficial [9], [10].

Multiple lines of evidence indicate that honey bees exhibit immunosenescence at the systemic level as they age and after they transition from nurses to foragers. The insect immune system can be categorized into mechanisms operating at barrier epithelial surfaces and at the systemic level. Barrier immunity refers to the cells and molecules that withstand pathogens on epithelial surfaces (*i.e*., outside the organism) while systemic immunity refers to the cells and molecules responsible for defending against pathogens that cross this barrier and therefore reside inside the organism [11], [12]. Systemic immunity in insects can be further categorized into two components: cellular and humoral [11], [12]. Examination of age-dependent or stage-dependent changes in the immune function of bees has focused on these two arms of systemic immunity. Most studies of the hemocytes demonstrate decreased numbers as bees age or transition between temporal castes [13], [14], [15], [16], [17], [18] and one type of cellular reaction, known as encapsulation, has been shown to be reduced in older bees [13]. Finally, fat body quantification [14], [15] has demonstrated stage-related reductions in this organ, which represents the major source of hemolymph immune effector proteins [12]. In correlation, inducible antimicrobial activity in the hemolymph, mostly produced by the fat body, also decreases with age [16]. However, not all systemic immune aspects decrease with age. For example, the humoral phenoloxidase-based melanization response does not diminish with honeybee age [17], [18], [20]. Thus, factors other than colony-level resource allocation may influence immune function in older bees.

As suggested above, the potential remaining intergenerational transfers of foragers are likely reduced due to their increased rate of mortality. However, foragers mediate a major mechanism of horizontal pathogen transmission between colonies [17], [18], [19] and from contaminated forage in the environment [20], [21]. As pathogen load is a primary determinant of disease spread within a group [22], immunosuppressed foragers would likely increase the acquisition of pathogens from the environment and subsequent transmission to the wider group population, potentially acting as a ‘super-shedder’ [23] as described by the ‘exposure risk hypothesis’ [24]. Therefore, a balance likely must be struck between the opposing objectives of individual pathogen resistance in older bees and allocation of limited colony-level resources to these individuals.

One potential route to balancing the above goals might be the maintenance of barrier immunity in aged individuals concomitant with the reduction in select systemic immune mechanisms. While a historical focus on systemic responses has existed in the study of immune responses in insects and other animals, recent efforts have shed new light on the importance of tissue specific responses in the expulsion of pathogens [25] especially in the context of barrier immune function.

We sought to extend observations of immunosenescence in honeybees to novel immune components in the context of barrier immunity at the epithelial surface of the digestive tract. Antimicrobial peptides (AMPs) represent one of the major effectors of innate immunity conserved throughout the animal kingdom [11], [12]. These small cysteine-rich cationic peptides act through altering microbial membrane properties [26] and intracellular metabolic processes [27]. They are thought to play a major role in barrier immunity as well as the systemic in insects. Many AMPs are regulated primarily at the transcript level for immediate translation and release. Others are known to be constitutively transcribed and translated, but for continual release or stored in peptide form for inducible release [27]. Honeybees possess six AMPs; *Abaecin, Hymenoptaecin, Apidaecin, Defensin 1*, *Defensin 2*, and *Apisimin* (reviewed in [28]). As five of these six AMPs are regulated at the transcriptional level in response to various pathogens [28], [29], [30], [31], we focused on transcript analysis for our study. The most well characterized transcriptional regulators involved in activation of these immune genes are the NF-κB-like proteins, Relish and Dorsal, that act downstream of the Imd and Toll signaling pathways, respectively [11], [12].

We observed that transcript levels of antimicrobial peptides (AMPs) measured in honey bee midguts did not follow a life stage-dependent decrease. None of the six known AMPs decreased when comparing nurses and foragers. When levels of AMPs are examined in the context of microbe levels, a number of microbe-associated changes in AMPs levels are observed which do not differ between nurses and foragers. These results demonstrate that one class of conserved barrier immune mechanisms does not undergo immunosenescence. These findings favor a model in foragers whereby effectors of the barrier immune system may be maintained while those of the systemic immune system are weakened.

## Materials and Methods

### Ethics Statement

All necessary permits were obtained for the described field studies. Honey bees were housed on private land for which research permission was granted by the owner.

### Honey bee Tissue Collection

Honey bees were collected from outbred colonies in Williamstown, Massachusetts, consisting of a typical mix of *Apis mellifera* subspecies found in North America, over two years at different times during the months of July-September. Only visibly healthy bees were collected, and all source colonies were visually inspected for symptoms of common bacterial, fungal, and viral diseases of honey bees. Nurses and foragers were collected from the same colonies at identical times using a mouth aspirator. Nurses were collected from the brood area of frames and were identified as nurses after they repeatedly placed their heads into honeycomb cells containing larvae. Foragers were collected from the front of the hive or on the landing board as they returned to the hive with visible loads of pollen on their legs [32]. Gut tissue was removed from abdomens and midguts were dissected and set aside for gene expression analysis. In select cases, the remaining abdominal wall was also independently used for gene expression analysis. All dissected material was placed into RNAlater (Invitrogen, San Diego, CA) for storage prior to analysis of individual workers' AMP expression.

### RNA Isolation, reverse-transcription and quantitative PCR for Gene Expression Analysis

RNA was prepared from bees from the described populations by manually crushing the tissue of interest with a disposable pestle in Trizol Reagent (Invitrogen, San Diego, CA) and extracting the RNA as per the manufacturer's instructions. RNA was subsequently DNAseI treated by RQ1 RNase-Free DNase (Promega, Madison, WI) and quantified. cDNA was synthesized using approximately 1 μg of RNA with the iScript cDNA Synthesis Kit (Biorad, Hercules, CA). Typically, 1 μl of cDNA was then used as a template for quantitative PCR to determine the levels of expression of genes of interest using the iQ SYBR Green Supermix (Biorad, Hercules, CA) in an iCycler thermo-cycler (Biorad, Hercules, CA). Primer sequences for transcripts of these AMP genes, as well as for the reference genes *β-actin* and *Rps5*, were from [28]. Primer sequences for the reference genes *Gapdh* were from [33]. Primer sequences for measurement of transcripts of the *Vitellogenin* and *Insulin Receptor* genes were from [34]. Primer sequences for *N. ceranae* and DWV [35], *C. mellificae* [36], and all bacteria [37] were previously reported. The difference between the threshold cycle number for *β-actin* and that of the gene of interest was used to calculate the level of that gene relative to *β-actin* using the ΔΔC_T_ method.

#### Statistical Analysis

Expression values were log10 transformed. Data is presented as boxes and whiskers and show 1st and 3rd interquartile range with lines denoting medians. Whiskers encompass 95% of the individuals. Outliers are denoted with filled circles, but were not removed in subsequent analyses. In some cases medians are plotted with error bars showing interquartile range. Data was compared using unpaired t-tests with Welch's correction when values fit normal distributions or Mann-Whitney U nonparametric tests when they did not fit normal distributions. Normality was assessed using Shapiro–Wilk tests. Differences in variance between the groups that fit normal distributions were assessed using an F-test. For correlations, Spearman's correlation analysis was used for comparing normally distributed populations and Pearson's correlation analysis was used for comparing non-normally distributed populations. For comparing the slopes of multiple lines, analysis of covariance (ANCOVA) was used.

## Results

### Sampling of honey bees reveals a broad range of AMP production in gut epithelium

**Figure 1:**
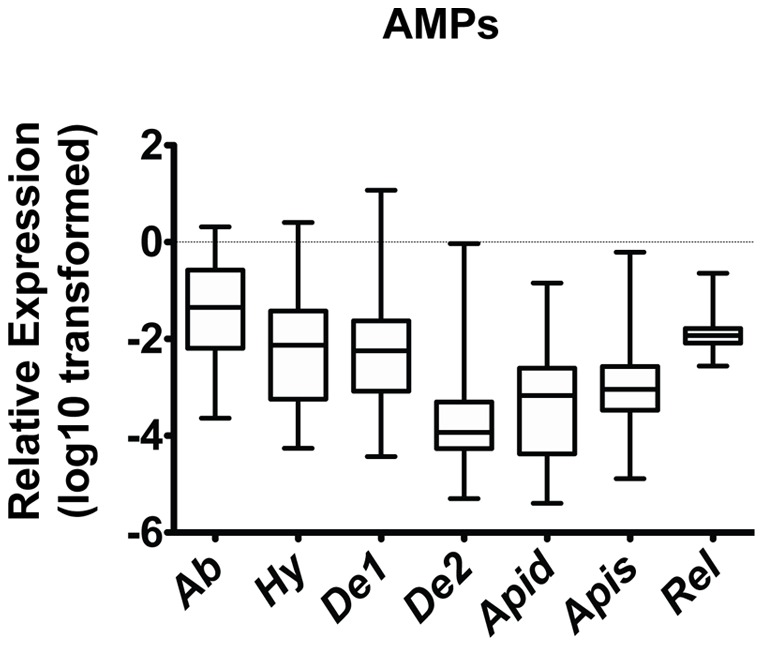
Broad range of AMP production in midgut barrier epithelium. Individual levels of the six honey bee AMPs (*Abaecin (n = 91), Hymenoptaecin (n = 91), Defensin 1 (n = 92)*, *Defensin 2 (n = 91), Apidaecin* (*n = 92)*, and *Apisimin* (*n = 92)*) and the immune signaling protein *Relish* (*n = 91)* relative to *β-actin* in midgut tissue from both nurses and foragers from multiple hives. Boxes show 1st and 3rd interquartile range with line denoting medians. Whiskers encompass 95% of the individuals. Outliers are denoted with filled circles.

Sampling of AMP gene expression in honeybees from healthy colonies revealed that individual bees display a remarkably broad range of AMP transcript levels in midgut tissue (Figure 1). This phenomenon was observed consistently over the six independent trials during two seasons (Supporting Figure S1B–G.). While *β-actin* has been used as a reference gene in a large number of studies in honey bees and has specifically been validated as a stable gene in two different studies [38], determining its stability in our hands was important in light of the range individual AMP levels observed above. We found similar Ct values for different individuals between trials for *β-actin* (Supporting Figure S1A). We also tested the stability of *β-actin* and two additional internal standard genes, *Gapdh* and *Rps5*, for trial 4 (Supporting Figure S2A). Again, the transcript levels of these ‘housekeeping‘ genes were remarkably consistent between identically treated samples. Finally, similar results were found using these three reference genes for the data set from one trial (Trial 4) (Supporting Figure S2B–G). Thus, these results have strengthened our confidence in the accuracy of our experimental strategy. Moreover, these results indicate that AMP genes can be expressed over a strikingly wide range in the midgut tissue of individual bees, consistent with the model that transcriptional regulation in the midgut is important for controlling their biological activity. Compared to the variation in AMP expression between individuals, we observed substantially less variation in the expression of the immune signaling protein *Relish* in all honeybees examined (Figure 1). Mean *Relish* expression was also very similar between trials (Supporting Figure S1B–G).

### Nurse and Forager Stages possess similar midgut AMP expression patterns

To examine effect of the nurse to forager transition on barrier AMP expression, we first used behavioral cues to classify sampled bees as nurses or foragers, as characterized previously [32]. We then used fatbody *Vitellogenin* and *Insulin Receptor* expression (both normally distributed after transformation) as an additional method for confirming our classification of each honeybee life stage [8]. As expected, foragers had decreased levels of *Vitellogenin* (p = 0.0021) and increased levels of *Insulin Receptor* expression (p = 0.031) (Supporting Figure S3A, B) in abdominal tissue.

**Figure 2:**
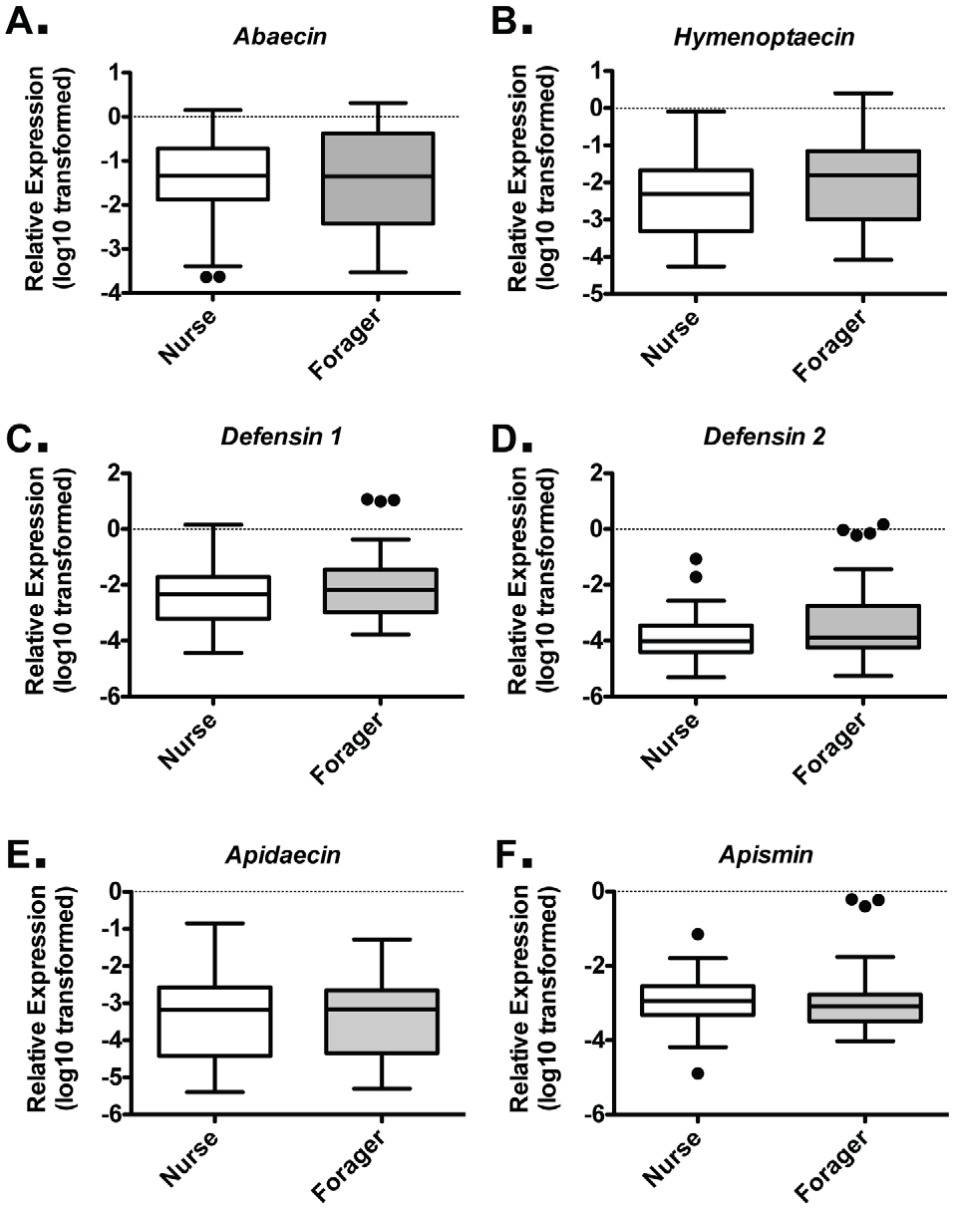
Similar midgut barrier AMP expression in nurse and forager stages. Individual levels of the six honey bee AMPs relative to *β-actin* in midgut tissue for Nurses (n = 46) and Foragers (n = 45), as assessed by behavior and location cues, for *Abaecin* (A), *Hymenoptaecin* (B), *Defensin 1* (C), *Defensin 2* (D), *Apidaecin* (E), and *Apisimin* (F) from the six trials. Boxes show 1st and 3rd interquartile range with line denoting medians. Whiskers encompass 95% of the individuals. Outliers are denoted with filled circles.

When comparing nurses and foragers, no difference in mean level of transcript abundance was found among all six AMP genes (Figure 2A–F). AMPs exhibit a wide range of expression levels in this tissue (Fig. 1), possibly due to individuals with induced and uninduced expression levels, as observed in other insects [11], [12]. To determine the stability of our reference gene between these two stages, we examined the consistency of Ct values for *β-actin* and two additional internal standard genes, *Gapdh* and *Rps5*, for nurses and foragers from trial 4 (Supporting Figure S2A). Any differences between the nurse and forager groups for these three independent reference genes were modest, and the relative AMPs expression levels for this trial were similar, regardless of which reference gene was used (Supporting Figure S2B–G). These results suggest that any variance is due to biologically relevant factors, such as pathogen load. Thus, we examined the variance in expression levels of the different AMPs using the F-test when the data were normally distributed. The range of expression levels did not differ for *Abaecin*, *Hymenoptaecin*, or *Apidaecin* between nurses and foragers, implying similar ability to induce these AMPs in both life stages. *Defensin 1, Defensin 2*, and *Apisimin* values were not normally distributed, and variance was not compared.

### Maintenance of AMP-inducing Signal Transduction Components in Foragers

**Figure 3:**
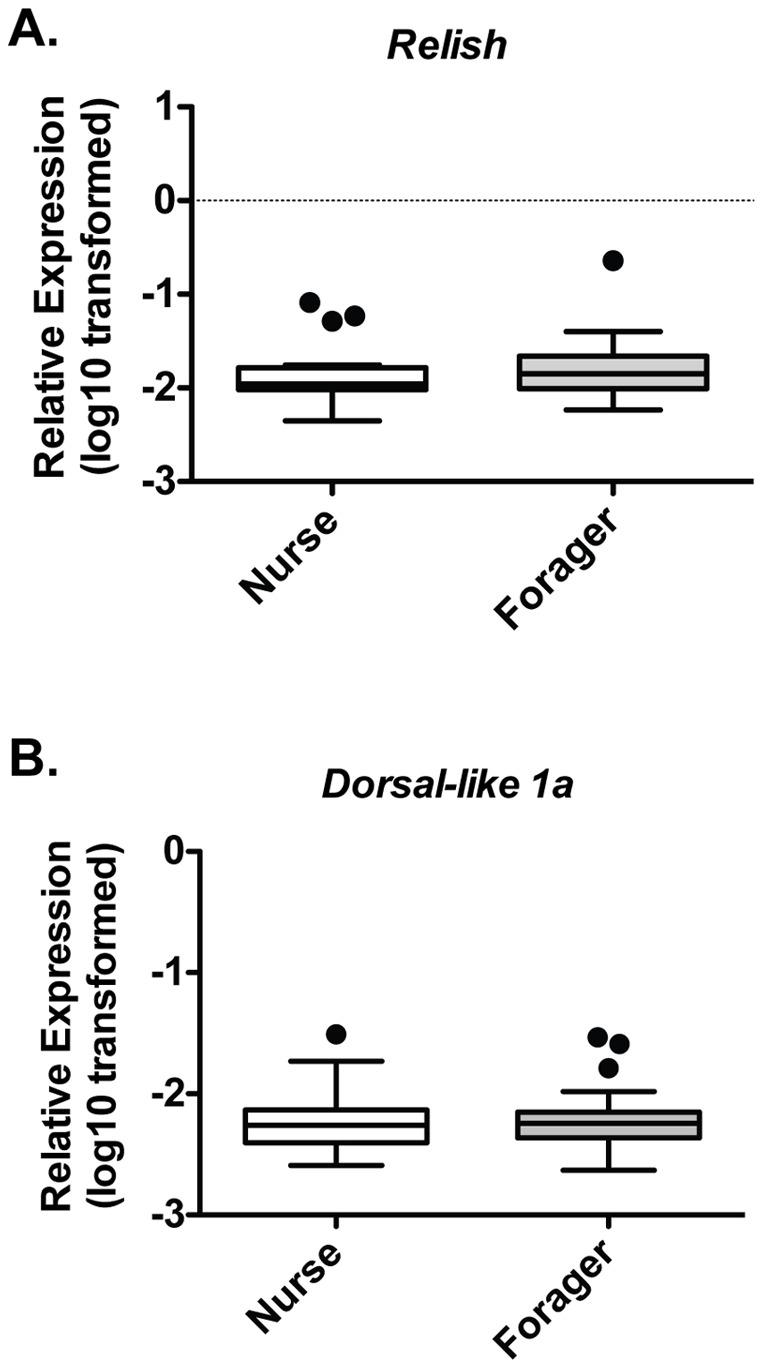
NFκB-family transcriptional activators of the Toll and Imd pathways are similar in nurses and foragers. Individual levels of *Relish* (A) and *Dorsal-like 1a* (B) relative to *β-actin* in midgut tissue from Nurses (n = 23) and Foragers (n = 22) from trials 1, 3, and 5. Boxes show 1st and 3rd interquartile range with line denoting medians. Whiskers encompass 95% of the individuals. Outliers are denoted with filled circles.

We also examined the maintenance of the immune signaling molecules Relish and Dorsal between nurses and foragers. We observed less variation in the expression of each of these two signaling components compared to that of the AMPs measured. These findings were consistent with our mechanistic understanding of these immune pathways, wherein the activity of the intermediate signaling components was predominantly regulated at the post-transcriptional level while transcriptional regulation contributed significantly to controlling effector levels [11], [12]. Mean transcript levels of Relish and both Dorsal-like proteins were not normally distributed and did not differ between the midguts of nurses and foragers (Figure 3A, B).

### Systemic AMP Expression in Nurses and Foragers

**Figure 4:**
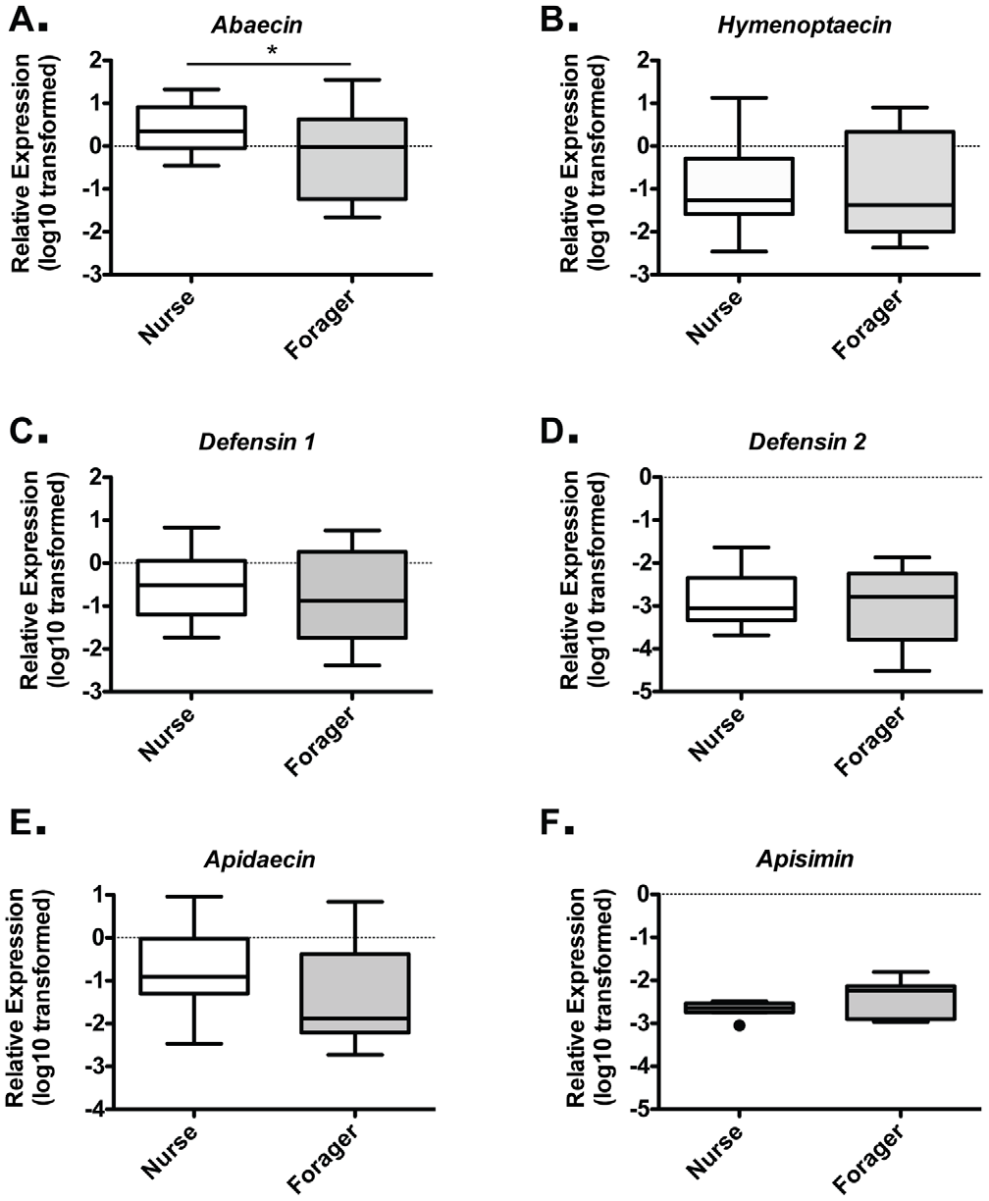
Nurse and forager AMP expression in fatbody tissue. Individual levels of the six honey bee AMPs relative to *β-actin* in abdominal tissue for Nurse and Foragers, as assessed by behavior and location cues, for *Abaecin* (A), *Hymenoptaecin* (B), *Defensin 1* (C), *Defensin 2* (D), *Apidaecin* (E), and *Apisimin* (F) from trials 3 and 4. Boxes show 1st and 3rd interquartile range with line denoting medians. Whiskers encompass 95% of the individuals. Outliers are denoted with filled circles.

In honey bee systemic immunity, antimicrobial activity has been shown to decrease in the hemolymph of foragers relative to nurses. However, the levels of individual AMPs at either the protein or transcript levels were not examined in these studies. As AMPs are produced in the fat body and their expression levels can be induced to increase the hemolymph levels of these proteins, we examined AMP expression in the abdomen, containing the fat body, at both stages. We found that only *Abaecin* showed a statistically significant decrease (p = 0.043) in average expression levels between nurses and foragers (Figure 4A). These results imply that, with the exception of *Abaecin*, AMP genes are transcribed at similar levels on a per cell basis in the fat body in both stages. However, in the context of reduced fat body mass (and presumably cell number) reported previously [14], [15], these results are consistent with a loss of systemic AMP production and reduced levels in the hemolymph.

### AMP and Microbe Correlation in Nurses and Foragers

We hypothesized that exposure to different pathogens over the course of the season played a critical role in the range of AMP expression observed in our study. The level of any immune parameter is always influenced by the immune competence of the organism and the level of any microbes that might induce an increase in the parameter. This is especially important in ecological immunology where there is limited ability to control the levels of various microbes in environmentally exposed populations [39], [40]. To begin to correlate microbe levels and immune parameters in our study, we examined the levels of microbe-specific RNA from various known honey bee pathogens [35], including *Nosema apis, Nosema ceranae, Ascosphaera apis, Crithidia mellificae*, DWV, BQCV, SBV, *Paenibacillus larvae*, and *Melissococcus plutonius* in pooled samples from all trials (data not shown). In addition to known pathogens, we examined levels of all bacteria and all fungi under the assumption that some microbes that are not overtly pathogenic could cause immune activation. Only levels of species-specific transcripts were detectable from *Nosema ceranae*, *Crithidia mellificae*, DWV; and transcripts representing all bacteria were detected at robust levels that differed among trials. For these microbes, we examined levels of microbe-specific RNA in individual bees.

**Figure 5:**
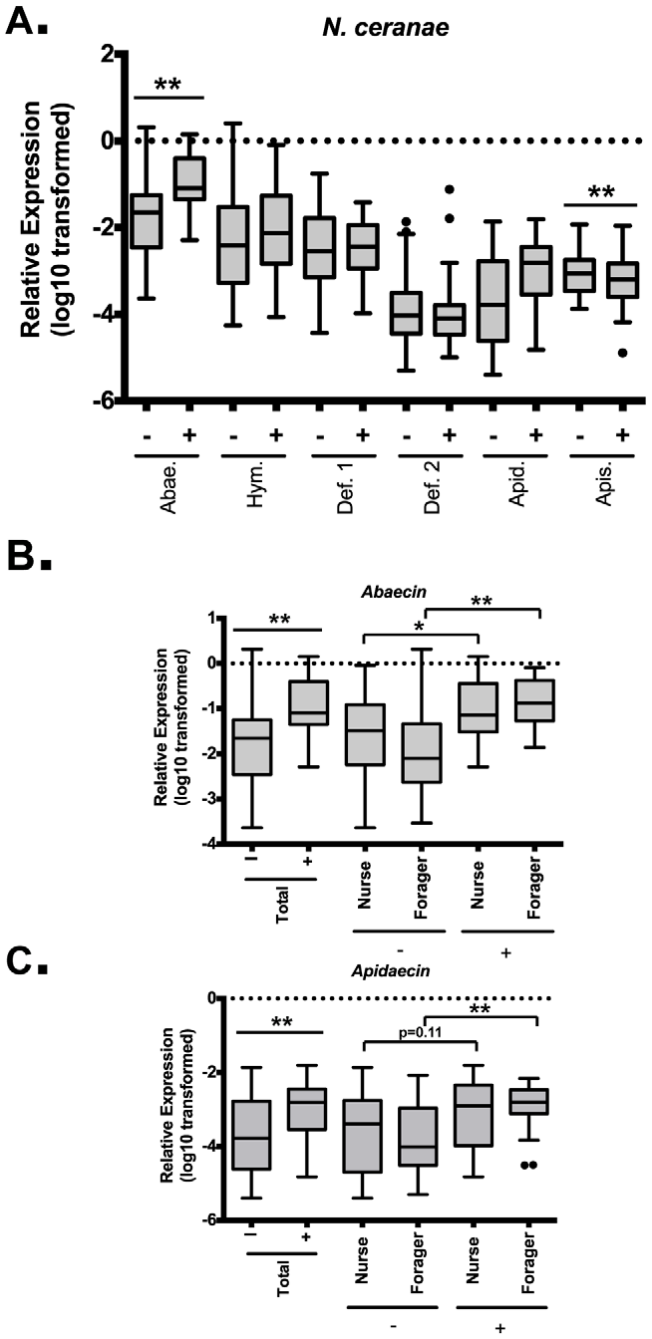
Differences in AMP expression between *Nosema* infected and non-infected bees are similar in nurse and foragers. Levels of the six honey bee AMPs (*Abaecin* (n = 91), *Hymenoptaecin* (n = 91), *Defensin 1* (n = 92), *Defensin 2* (n = 91), *Apidaecin* (n = 92), and *Apisimin* (n = 92) relative to *β-actin* in midgut tissue from bees that were positive (+) or negative (−) for *Nosema ceranae* (A). Levels of *Abaecin* (B) and *Apidaecin* (C) relative to *β-actin* in midgut tissue from bees that were positive (+) or negative (−) for *Nosema ceranae* for all bees and split into nurses and foragers. Boxes show 1st and 3rd interquartile range with line denoting medians. Whiskers encompass 95% of the individuals. Outliers are denoted with filled circles.

Levels of *N. ceranae* were not detected in individual bees from trial 1 through trial 4, but were detected at variable levels in individual bees from trials 5 and 6 (Supporting Figure S4A). We classified individual bees from these trials into *N. ceranae* positive and *N. ceranae* negative groups and examined AMP expression levels in the two groups (Figure 5A). We found that only *Abaecin* (p<0.0001) and *Apidaecin* (p = 0.0005) differed between the groups, with both being increased ∼ 10-fold in *N. ceranae* positive bees. When the results were examined for nurses and foragers (Figure 5C), we found significant increases in *Abaecin* for nurses (p = 0.037) and foragers (p<0.0001) and in *Apidaecin* for foragers (p = 0.0017). *Apidaecin* demonstrated a trend in nurses (p = 0.11) for a difference in expression for in *N. ceranae* positive and negative bees. There was not a statistically significant difference in the levels of either *Abaecin* or *Apidaecin* between nurses and foragers in the *N. ceranae* positive or negative groups. The levels of *N. ceranae* in trials 5 and 6 were not different between nurses and foragers (data not shown). These results suggest that bees respond to the presence of *N. ceranae* by increasing the transcription of *Abaecin* transcription and that the ability to induce expression is not affected by the transition to foraging.

Levels of *C. mellificae* were detected at variable levels in individual bees from (Supporting Figure S4B). We examined AMP expression among groups positive or negative for *C. mellificae* (Supporting Figure S5A). We found that only *Apidaecin* (p = 0.027) differed between the groups, exhibiting a ∼ 5-fold decrease in *C. mellificae* positive bees. The levels of *C. mellificae*-positive bees were much higher in foragers relative to nurses (p = 0.0014) (Supporting Figure S5B), thus precluding analysis of *Apidaecin* between these groups.

**Figure 6:**
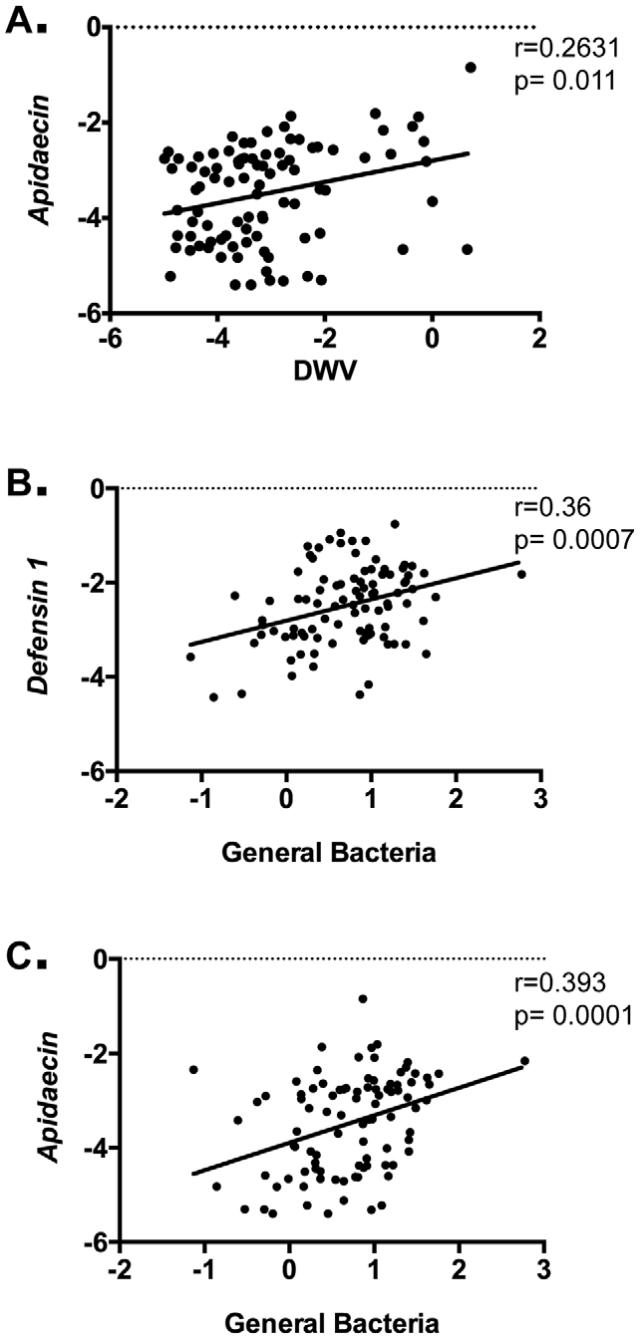
Differences in AMP expression based on level s of DWV and of all bacteria are similar in nurse and foragers. Correlation of levels of *Apidaecin* (n = 92), with levels of DWV (A) and *Defensin 1* (B) and *Apidaecin* (C), with levels of all bacteria (16S RNA).

DWV was detected in all bees from trial 1 through trial 6 (Supporting Figure S4C), in line with previous studies showing high prevalence of this virus [41]. To determine how levels of this virus affected AMP expression, we performed regression and correlation analysis of all AMPs versus DWV. We found that only *Apidaecin* (p = 0.011, Spearman correlation r = 0.2631) demonstrated a significant linear correlation with DWV (Figure 6A). When the results were compared between nurses and foragers, no significant difference was found in the slopes or intercepts of these two lines, suggesting that the response of both nurse and forager bees to DWV are equivalent. The levels of DWV were not different between nurses and foragers (data not shown).

Variable levels of total bacteria were found in all bees (Supporting Figure S4D), in agreement with recent studies demonstrating a stable commensal population as well as the presence of other non-commensal species [42], [43], [44], [45]. Regression and correlation analysis of all AMPs versus total bacteria revealed positive relationships with *Defensin 1* (p = 0.0007, Pearson correlation r = 0.36) (Figure 6B) and *Apidaecin* (p = 0.0001, Spearman correlation r = 0.393) (Figure 6C). When the data were stratified into nurse and forager groups, we found no significant difference in the slopes or intercepts, suggesting that the responses of both groups to bacteria levels are equivalent. The levels of total bacteria were not different between nurses and foragers (data not shown).

## Discussion

There is growing interest in the study of barrier immunity, as its role in pathogen defense has become more appreciated [25]. To our knowledge, this study is the first examination of barrier immunity in the adult honey bee. We noted a remarkable range of AMP expression levels in the barrier epithelia of the digestive tract in individual bees, consistent with the robust induction of these genes in other insects [11], [12]. We also observed differences in mean colony expression levels of the various AMPs from trial to trial (Supporting Figure S1B–G).

We reasoned that exposure to different pathogens over the course of the season leads to a differential induction of AMPs that results in the broad range of expression levels observed. In fact, we found that the levels of a number of microbes were varied over different trials, in agreement with other studies [36]. Furthermore, we were able to discern positive relationships between microbe levels and the transcript abundance of specific AMPs, key immune effector genes. Importantly, these associations were not found to differ between nurses and foragers, indicating that the ability to increase or decrease the expression of immune genes in response to microbes was not compromised by this transition. Our data provide the first evidence of inducible AMP expression in the barrier immune system of the honey bee. Specifically we found a positive association between *N. ceranae* levels and the quantity of two AMPs, *Abaecin* and *Apidaecin*. Our findings differ from a recent study examining gene expression changes in the midgut in response to *N. ceranae* infection, where no changes in AMP expression were observed [46]. A possible explanation for the differences is that this study examined a single time-point, 7 days after infection, which may have missed early induction of immune genes before larger changes in midgut tissue occurred. We also found a positive correlation between the relative amount of DWV and the AMP *Apidaecin*. No studies to date have examined immune response to virus infection in the midgut epithelia of honey bees, and future studies will be required to ascertain the biological significance of this finding.

We also found strong positive correlation between the amount of total bacteria and transcript levels of two AMPs, *Defensin 1* and *Apidaecin*. This result is especially interesting in light of recent studies showing a highly stable commensal population in honey bees as well as a number of species that appear more variable [43], [44]. It is appealing to speculate that more detailed analyses of these bacteria and their consequent induction of AMP expression will uncover novel relationships between bacteria species and the honey bee. Notably, different microbes are associated with different profiles of AMP genes change in the midgut of the honey bee. This agrees with our current understanding of immunity in another model insect, *Drosophila melanogaster*, where AMPs possess distinct activating pathways and target specificities [26], [47].

It is unlikely that all relationships between various microbes and AMP transcriptional output were uncovered by this analysis. First, we used a candidate approach to examining specific pathogens and did not include some viruses and other microbial pathogens that may infect honey bees through their digestive tract [1]. In addition, as our protocol did not use the ideal extraction methods for recovery of the contents of certain types of bacteria with thick cell walls, such as gram positive species, it is likely that our examination of total bacterial levels is incomplete. It is also likely that some relationships exist between subgroups of bacteria and immune gene induction that would not be appreciated when examining total bacteria as opposed to specific subspecies. Finally, co-infection by multiple species is likely to have additive and non-additive effects on immune gene transcription that would complicate analysis. For example, we found that the amount of total bacteria was greater in *N. ceranae* positive bees than in those that did not have *Nosema* by a factor of >3-fold (p = 0.0268) (Supporting Figure S5C). Co-infection by multiple microbes is rampant in wild populations [48] and the effect this has on barrier immune activation in insects is incompletely understood [49]. Co-infections, however, have significant implications for individual bees and overall colony health [50], and future studies to examine these relationships are warranted. In addition to microbe levels, other factors such as seasonal changes in forage and the genetic diversity both within and between colonies likely also play a role, as these trials were done on different hives over the course of more than a year.

Studies of immunosenescence have largely focused on two aspects of systemic immunity: either cellular or humoral mechanisms. Cellular immunity declines with age in most invertebrate models examined to date [55], [56], [57] including honey bees [13], [14], [15], [16], [17]. Studies examining the effects of aging on humoral systemic immunity in insects reveal a complex picture. Age-dependent loss of phenoloxidase activity, one humoral immune effector, appears to be species-specific, occurring in bumble bees, but not honey bees [17], [18]. Baseline levels of systemic AMPs increases with age in *Drosophila*, while AMP induction apparently decreases [58], [59], [60]. Similarly, unchallenged or aseptically challenged honey bee workers display an increase in AMP activity with age, however ability to induce systemic AMP activity in the hemocoel in response to LPS is reduced [16]. Survival in response to systemic bacterial infection in *Drosophila* also decreases with age, while bacterial clearance ability does not, implying higher rates of immune activation related damage [51]. At the systemic level, similar observations are seen in mammals; immune function declines, baseline activation increases, and immune-associated pathogenesis increases [10], [52].

Much less is known about immunosenescence in the setting of barrier epithelia in any species [53]. While other studies have begun to address immunosenescence in barrier epithelia of the digestive tract in other invertebrate species [54], [55], [56], no data currently exists for effector mechanisms in this setting. Immunosenescence may affect barrier and systemic defenses differently. For example, a continued investment to barrier immunity may be especially critical in the digestive tract where immune–mediated maintenance of a functional, commensal, microbial niche may be as important as pathogen resistance [57], [58]. A specific gut-associated microbiome has been established for honey bees [42], and differences in larval and adult commensal species has been observed [44], [59], [60]. In addition to understanding protective value, insight into the relative costs of immunity at the barrier and systemic levels, and for different mechanisms within each level, may be critical for understanding the differential effects of immunosenescence on these systems. Studies designed to measure the true costs of immune activation, such as measuring how oral or systemic infection alters nutrient storage [61] could provide insight into this question.

The awareness that the nurse to forager transition is not absolute in honey bees has important significance for the study of immunosenescence reported here. First, foragers may revert to nurse-like physiology and behavior in response to demographic changes [6]. Second, honey bee workers may also develop into long-lived, diutinus workers or ‘winter bees’ instead of transitioning to foragers. While workers in this state share many of the physiological and behavioral characteristics of nurses, they are likely distinct and are critical in temperate climates for overwintering when brood production halts [62]. Understanding the impact of these two additional states on immune function will be important for understanding the relative importance of temporal and behavioral/physiological age on immunosenescence. In the case of forager to nurse reversion, it has been shown that reversal of some aspects of systemic immunosenescence occurs [63]. Immune function has not been studied in depth in diutinus bees. However, senescence-associated defects in the function of other physiological systems in forager bees can be forestalled for months by entry into this state [64]. Future study into the prevalence of immunosenescence in both systemic and barrier immune systems in this class of bees is warranted. The nurse to forager transition is controlled by factors signaling the demographic need of the hive through effects at the level of the individual. At the physiological level, this appears to be mechanistically regulated by the Juvenile Hormone/Vitellogenin axis [62]. Vitellogenin itself represents a protein at the intersection of nutritional status and molecular control of the physiological and behavioral changes associated with the nurse to forager transition. Induction of systemic immunosenescence [65] and its reversal [63] appear to be controlled in part by levels of this protein. It will be interesting to dissect the molecular differences in the barrier immune function studied here that appear to render it impervious to changes in this important molecule.

We hypothesized that barrier immune function might be maintained in foragers to diminish pathogen spread, while systemic immune function would diminish as a function of foragers' decreasing remaining resource transfers to the colony. In agreement with this hypothesis, we do not observe a decrease in mean expression levels or variance in AMPs after the transition from nurse to forager. In addition, we do not observe differences in microbe-associated changes in gene expression. Furthermore, our results provide a more comprehensive view of immune function in honey bees by providing the first examination of barrier immune mechanisms. In addition, we provide evidence that microbe levels affect immune gene transcription in the barrier epithelia of this species in a manner similar to that observed in other species. None of the previous studies of barrier immunosenescence in invertebrates [54], [55], [56] have examined the effect on aging on AMPs, which represent a highly conserved and relevant immune mechanism in these tissues. Thus, these findings offer novel insight into the effects of immunosenescence on barrier immune function in insects more generally. The intergenerational theory of aging can explain immunosenescence as the consequence of reduced resource allocation to individuals with increased rate of mortality and fewer opportunities to perform resource transfers to the group. Reducing resource allocation to systemic immune mechanisms while maintaining those in barrier epithelium might be a reasonable strategy for balancing individual pathogen resistance and energy allocation for optimal colony-level vigor. However, our results suggest that a more comprehensive understanding of immune mechanisms, costs, and pathogen interactions will be required for full understanding of immunosenescence in honey bees and other social insects.

## Supporting Information

Figure S1 **Trial-dependent expression differences of AMPs.** Median threshold cycle (Ct) and interquartile range is shown for *β-actin* for trials 1–6 (A). Individual levels of AMPs and *Relish* relative to *β-actin* are shown for trials 1 (B), 2 (C), 3 (D), 4 (E), 5, and 6 (F). Values for individual bees are shown as circles. Symbol and error bars represent the Mean ± SEM.
(TIF)

Figure S2 **Trial 4 results compared using difference reference genes.** Median threshold cycle (Ct) and interquartile range is shown for *β-actin, Gapdh*, and *Rps5* for nurses and foragers for trial 4 (A). Individual levels AMPs relative to *β-actin, Gapdh*, and *Rps5* for *Abaecin* (B), *Hymenoptaecin* (C), *Defensin 1* (D), *Defensin 2* (E), *Apidaecin* (F), and *Apisimin* (G).
(TIF)

Figure S3 **Nurse and forager confirmation by molecular markers.** Individual levels of *Vitellogenin* (A) and *Insulin Receptor* (B) relative to *β-actin* in abdominal tissue from trials 3 and 4, and trial 4, respectively. Median threshold cycle (Ct) and interquartile range is shown. Statistical significance was assessed by unpaired t-tests with Welch's correction. *p<0.05 and **p<0.01.
(TIF)

Figure S4 **Microbe levels in individual bees.** Levels of *Nosema ceranae* (A) *Crithidia mellificae* (B), DWV (B), and all bacteria (D) relative to *β-actin* in midgut tissue from both nurses and foragers from multiple hives. Values for individual bees are shown as circles. Symbol and error bars represent the Mean ± SEM.
(TIF)

Figure S5 **AMP levels and** ***Crithidia.*** Levels of the six honey bee AMPs, *Abaecin*, *Hymenoptaecin*, *Defensin 1, Defensin 2*, *Apidaecin*, and *Apisimin* relative to *β-actin* in midgut tissue from bees that were positive (+) or negative (−) for *Crithidia mellificae* (A). Levels of *Crithidia mellificae* (B) relative to *β-actin* in midgut tissue from nurses and foragers. Levels of all bacteria (C) relative to *β-actin* in midgut tissue from bees that were positive (+) or negative (−for *Nosema ceranae*. Boxes show 1st and 3rd interquartile range with line denoting medians. Whiskers encompass 95% of the individuals. Outliers are denoted with filled circles.
(TIF)
