# Supplementary figures and images for: Barrier Immune Effectors Are Maintained during Transition from Nurse to Forager in the Honey Bee
Source: PLoS One. 2013 Jan 8;8(1):e54097. doi: 10.1371/journal.pone.0054097 (PMC3540063; doi:10.1371/journal.pone.0054097)

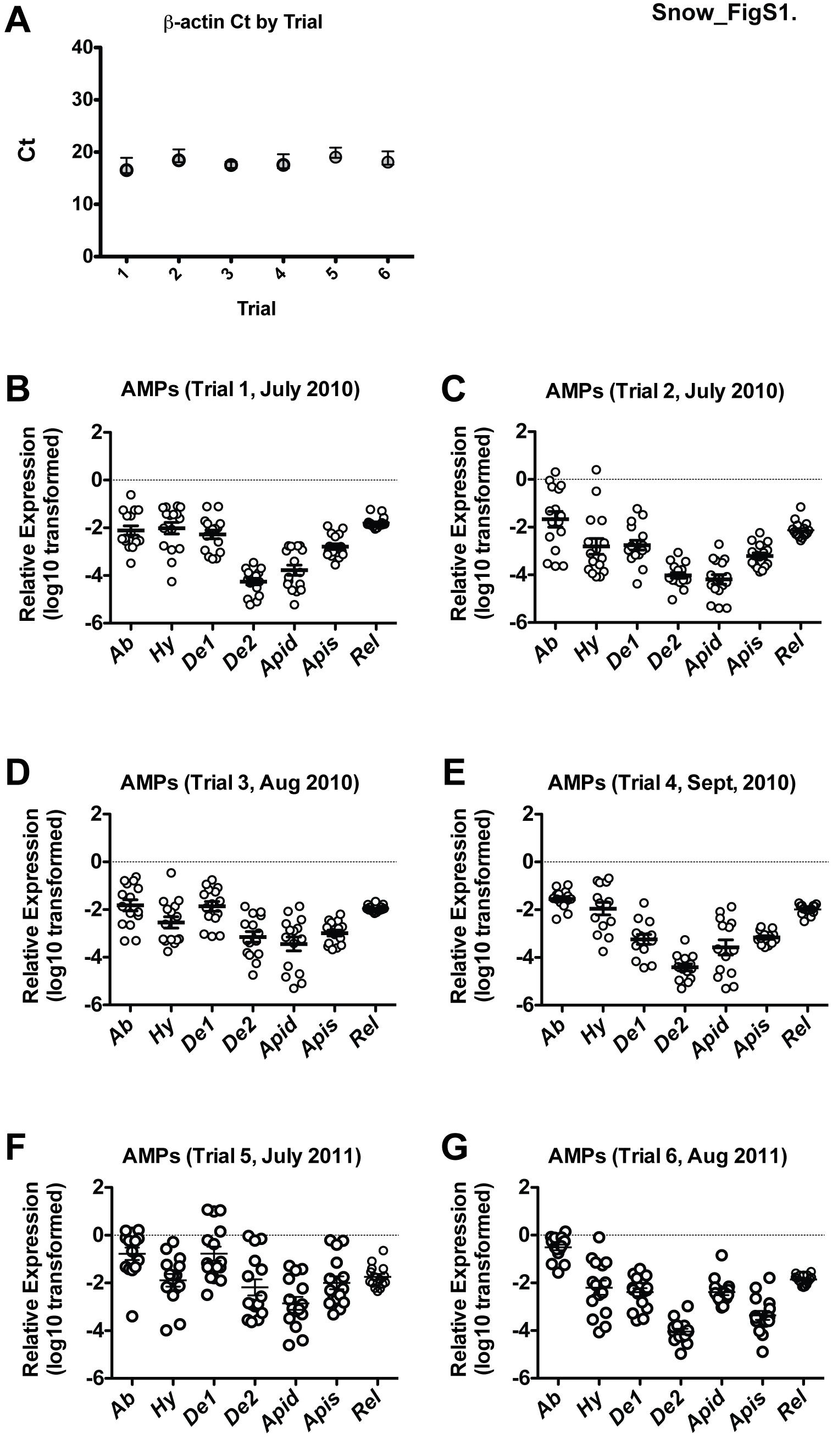

Supplement: Figure S1 — Trial-dependent expression differences of AMPs. Median threshold cycle (Ct) and interquartile range is shown for β-actin for trials 1–6 (A). Individual levels of AMPs and Relish relative to β-actin are shown for trials 1 (B), 2 (C), 3 (D), 4 (E), 5, and 6 (F). Values for individual bees are shown as circles. Symbol and error bars represent the Mean ± SEM. (TIF) [file pone.0054097.s001.tif]

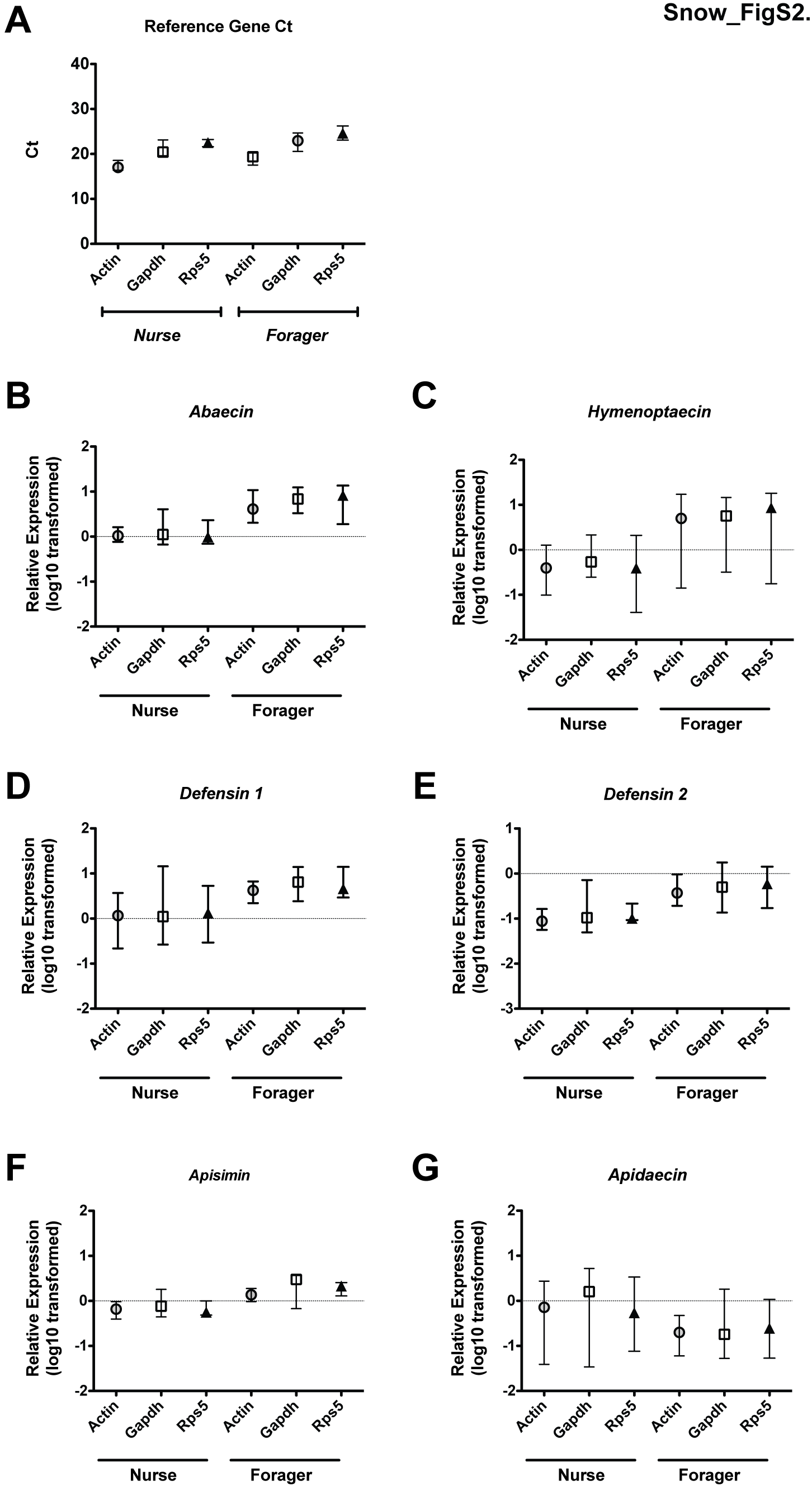

Supplement: Figure S2 — Trial 4 results compared using difference reference genes. Median threshold cycle (Ct) and interquartile range is shown for β-actin, Gapdh, and Rps5 for nurses and foragers for trial 4 (A). Individual levels AMPs relative to β-actin, Gapdh, and Rps5 for Abaecin (B), Hymenoptaecin (C), Defensin 1 (D), Defensin 2 (E), Apidaecin (F), and Apisimin (G). (TIF) [file pone.0054097.s002.tif]

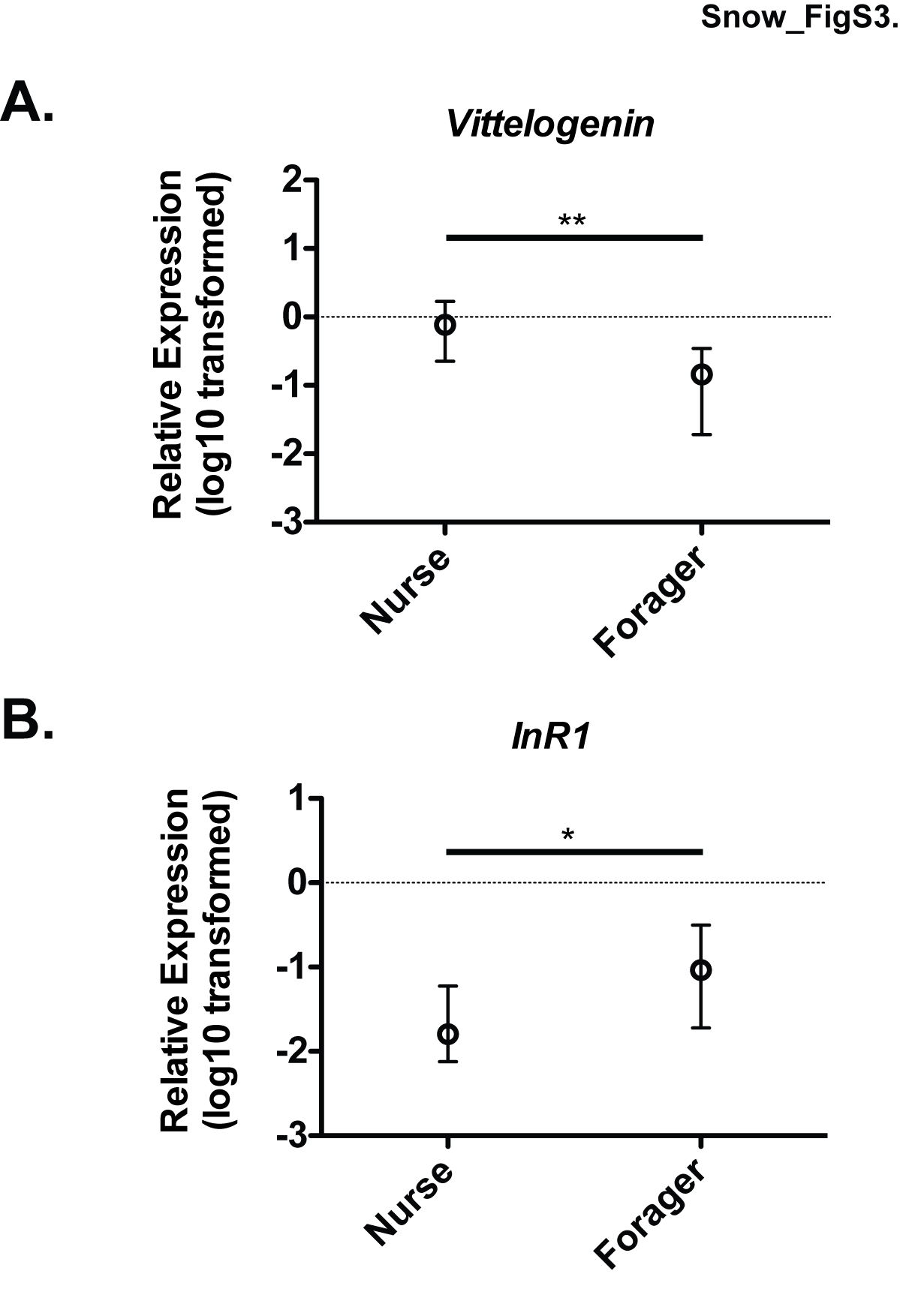

Supplement: Figure S3 — Nurse and forager confirmation by molecular markers. Individual levels of Vitellogenin (A) and Insulin Receptor (B) relative to β-actin in abdominal tissue from trials 3 and 4, and trial 4, respectively. Median threshold cycle (Ct) and interquartile range is shown. Statistical significance was assessed by unpaired t-tests with Welch's correction. *p<0.05 and **p<0.01. (TIF) [file pone.0054097.s003.tif]

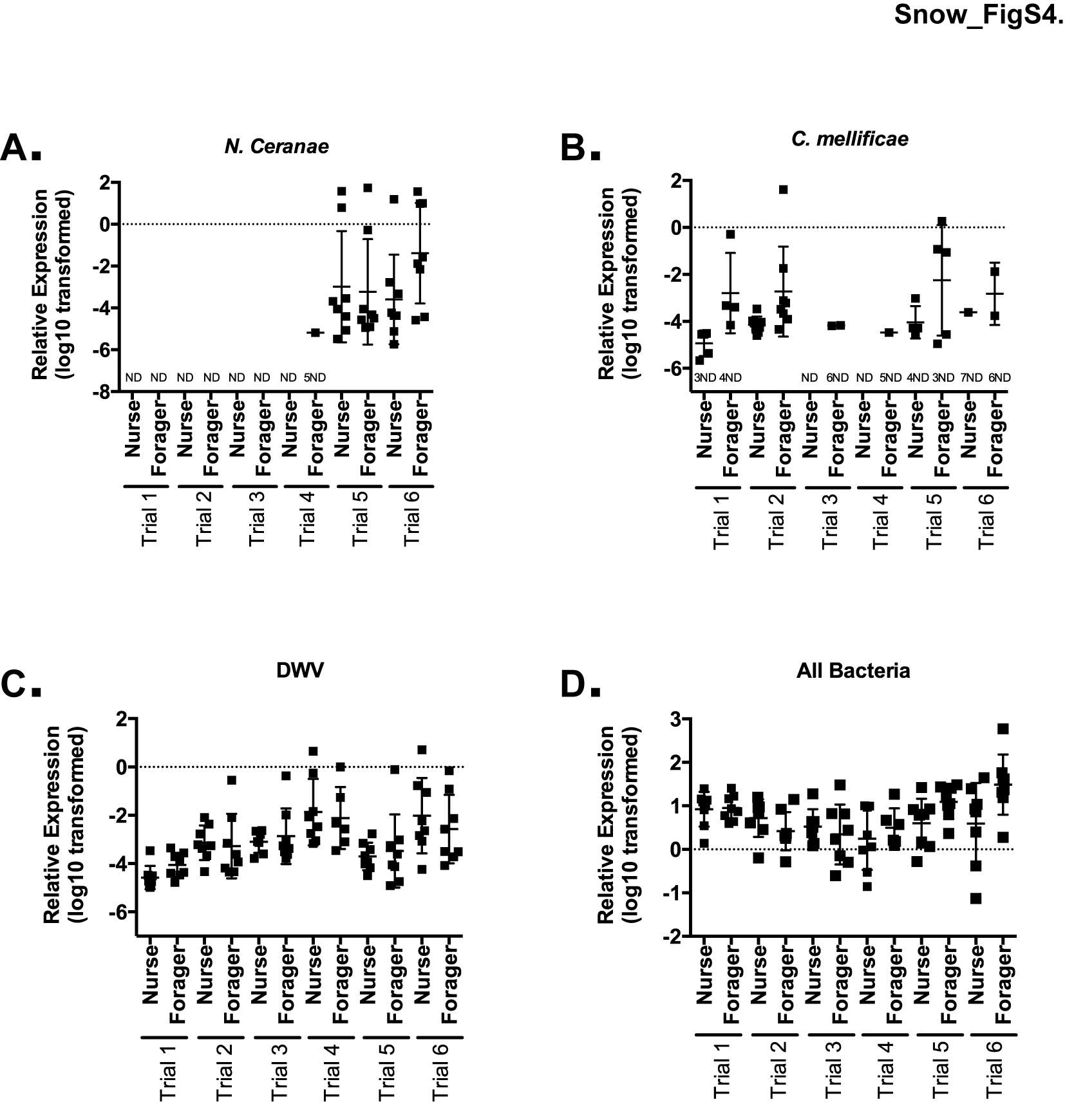

Supplement: Figure S4 — Microbe levels in individual bees. Levels of Nosema ceranae (A) Crithidia mellificae (B), DWV (B), and all bacteria (D) relative to β-actin in midgut tissue from both nurses and foragers from multiple hives. Values for individual bees are shown as circles. Symbol and error bars represent the Mean ± SEM. (TIF) [file pone.0054097.s004.tif]

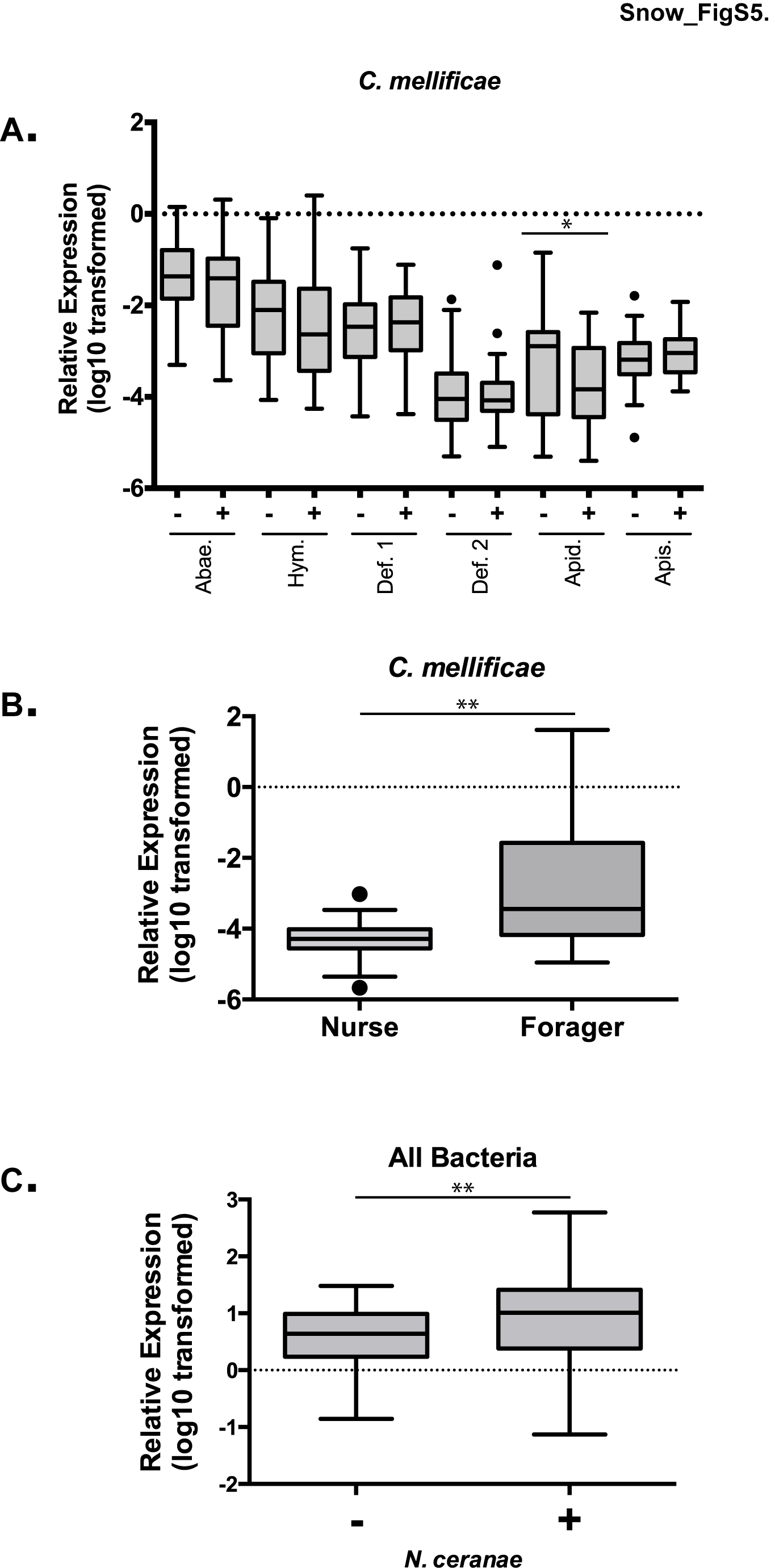

Supplement: Figure S5 — AMP levels and Crithidia. Levels of the six honey bee AMPs, Abaecin, Hymenoptaecin, Defensin 1, Defensin 2, Apidaecin, and Apisimin relative to β-actin in midgut tissue from bees that were positive (+) or negative (−) for Crithidia mellificae (A). Levels of Crithidia mellificae (B) relative to β-actin in midgut tissue from nurses and foragers. Levels of all bacteria (C) relative to β-actin in midgut tissue from bees that were positive (+) or negative (−for Nosema ceranae. Boxes show 1st and 3rd interquartile range with line denoting medians. Whiskers encompass 95% of the individuals. Outliers are denoted with filled circles. (TIF) [file pone.0054097.s005.tif]
